# Soil Moisture Content Retrieval from Remote Sensing Data by Artificial Neural Network Based on Sample Optimization

**DOI:** 10.3390/s22041611

**Published:** 2022-02-18

**Authors:** Qixin Liu, Xingfa Gu, Xinran Chen, Faisal Mumtaz, Yan Liu, Chunmei Wang, Tao Yu, Yin Zhang, Dakang Wang, Yulin Zhan

**Affiliations:** 1 Aerospace Information Research Institute, Chinese Academy of Sciences, Beijing 100094, China; liuqx@radi.ac.cn (Q.L.); guxf@radi.ac.cn (X.G.); chenxr@aircas.ac.cn (X.C.); faisal@aircas.ac.cn (F.M.); liuyan@aircas.ac.cn (Y.L.); wangcm@aircas.ac.cn (C.W.); yutao@radi.ac.cn (T.Y.); 2 University of Chinese Academy of Sciences, Beijing 100049, China; 3 School of Remote Sensing and Information Engineering, North China Institute of Aerospace Engineering, Langfang 065000, China; 4 Beijing Institute of Space Long March Vehicle, Beijing 100076, China; zhangyin@radi.ac.cn; 5 School of Environmental Science and Engineering, Southern University of Science and Technology, Shenzhen 518055, China; wangdk@aircas.ac.cn

**Keywords:** soil moisture content, artificial neural network, sample optimization, synthetic aperture radar, optical remote sensing image

## Abstract

Soil moisture content (SMC) plays an essential role in geoscience research. The SMC can be retrieved using an artificial neural network (ANN) based on remote sensing data. The quantity and quality of samples for ANN training and testing are two critical factors that affect the SMC retrieving results. This study focused on sample optimization in both quantity and quality. On the one hand, a sparse sample exploitation (SSE) method was developed to solve the problem of sample scarcity, resultant from cloud obstruction in optical images and the malfunction of in situ SMC-measuring instruments. With this method, data typically excluded in conventional approaches can be adequately employed. On the other hand, apart from the basic input parameters commonly discussed in previous studies, a couple of new parameters were optimized to improve the feature description. The Sentinel-1 SAR and Landsat-8 images were adopted to retrieve SMC in the study area in eastern Austria. By the SSE method, the number of available samples increased from 264 to 635 for ANN training and testing, and the retrieval accuracy could be markedly improved. Furthermore, the optimized parameters also improve the inversion effect, and the elevation was the most influential input parameter.

## 1. Introduction

The soil moisture content (SMC) refers to the volume of water present in the gaps between surface soil granules. The SMC is a critical parameter for investigating and predicting the factors associated with climate change. It also plays a key role in various fields of science such as ecology, hydrology, and agriculture [1,2,3]. However, the measurement and acquisition processes of SMC are pretty challenging. Although conventional measurement methods, such as time-domain reflectometry and gravimetric technique, may yield relatively precise SMC values at monitoring sites, they can hardly provide soil moisture information in the case of large areas, making it difficult to describe the spatial heterogeneity pattern of soils. In addition, such field measurements require a considerable workforce and lead to the deterioration of the local soil environment [4]. Remote sensing (RS) techniques have been rapidly developed in recent decades, featuring fast data acquisition and low effort consumption in their application to land surface investigation. Among other RS techniques, synthetic aperture radar (SAR) has been proven to be promising. Apart from such optical sensors, the SAR can collect ground surface information even at night and under cloudy weather conditions. The competitive penetrating power and the direct relationship between SMC and the SAR observations also make the estimation of SMC much more reliable [5]. Researchers have fully exploited this advantage; therefore, the SAR has been extensively employed for SMC retrieval [6,7,8,9].

Regarding microwave data, theoretical and semi-empirical models have been established for SMC estimation, such as the integral equation model (IEM) [10], advanced integral equation model (AIEM) [11], Oh model [12], Dubois model [13], Michigan microwave canopy scattering model (MIMICS) [14], water-cloud model (WCM) [15], and tau–omega model [16]. In applying these microwave models, considering the impact of land surface vegetation on microwave RS data [17], the effect of vegetation should be accurately quantified for a more precise SMC estimation. Because optical RS data are more sensitive to land surface vegetation, the combination of optical and microwave detection has emerged as an intuitive approach. Instead of deploying a single model for SMC retrieval, researchers have attempted to modify the original models by integrating them with optical information, hence carrying out tasks such as synergistic SMC inversion using both optical and SAR images [18,19,20,21]. Zhang et al. [22] built a radar backscattering coefficient database based on advanced integral equation model (AIEM) simulation, eliminated the vegetation effect using the WCM, and acquired the SMC by minimizing the difference between the observed bare soil backscattering coefficient and the simulated one. Han et al. [23] put forward a model-coupling method using GF-3 and GF-1 data by incorporating a series of models and achieved high-precision soil moisture mapping. Khabazan et al. [24] compared the capabilities of the IEM, Oh model, and Dubois model for surface soil moisture retrieval with C-band and L-band data to analyze the different conditions of vegetation land cover systematically. Overall, the application of theoretical and semi-empirical models can help represent the physical transmission processes more accurately. However, there are evident drawbacks. Most of the models stated above have complex structures and variables. Determining the values of some of these variables, such as the surface roughness and vegetation water content, requires laborious field experiments, and precise outcomes can hardly be ensured [23,25].

As a non-linear empirical model, the artificial neural network (ANN) can build an implicit relationship between input data and output targets, and it has been proven effective for SMC retrieval [26]. Studies on SMC estimation using ANNs with microwave and optical RS data have been conducted. For example, Baghdadi et al. [27] combined Radarsat-2 and Landsat data and inputted them to an ANN for simultaneous SMC and leaf area index estimation; the merits and demerits of radar data in dual- and full-polarization modes were also highlighted. El Hajj et al. [28] mainly focused on agricultural areas and depicted high-resolution SMC maps of bare and vegetation-covered farmlands using the backscattering coefficient and normalized difference vegetation index (NDVI) as input parameters. El Hajj et al. [29] combined “vegetation descriptors” derived from optical images and backscattering coefficients as ANN training and testing samples, and three different inversion configurations were compared in terms of their performances.

As we know, samples are the key elements of ANN. To obtain ideal retrieval results, both the quantity and quality of the samples for ANN training and testing should be guaranteed. That is to say, not only should the sample pool be large enough, but also the input parameters of the samples should be inclusive of the features that are helpful to accurate SMC retrieval. As for the quantitative optimization, sufficient samples are conducive to improving the training accuracy and representing various geographical situations [30,31,32]. In many previous studies, when choosing samples, it was often required that the data of each monitoring site in the entire research area be “complete” at one specific time, entailing remote sensing images and in situ measurements of sound quality [28,33,34,35]. However, such conditions are hard to meet.

For one thing, the use of optical images is associated with contamination from clouds, thick fogs, and mists [36], which may lead to a shortage of optical RS data. For another, there are temporal discrepancies between in situ measurements in a study area because instrument malfunctions make it impossible to acquire data of some parts of the monitoring sites in specific periods, which may also lead to the shortage of in situ data [37]. Therefore, gathering enough samples for ANN training and testing is difficult. As for qualitative optimization, it is of importance to determine the input parameters of ANN. In previous studies, some common variables, including the radar incidence angle, VH/VV backscattering coefficients, and NDVI, were investigated about the effectiveness of being used as inputs of the ANN for SMC retrieval [27,33]. In fact, in addition to these commonly considered ones, variables about other factors, such as local land use, topography, and phenology, can also be influential in local soil moisture and deserve to be given close attention.

To address the problem of quantitative optimization, a novel sparse sample exploitation (SSE) method was proposed, whereby a part of the samples that were otherwise excluded could be sufficiently utilized and incorporated into the SMC retrieval procedure. To address the problem of qualitative optimization, we extended the array of input parameters of ANN for SMC retrieval. Apart from the radar incidence angle, VH/VV backscattering coefficients and NDVI, which were included in this paper as the basic input parameters, parameters such as LST, land cover type, elevation, slope, and data acquisition time, are likewise considered as the inputs of ANN in this paper. The sensitivity of SMC retrieval to these parameters was discussed.

The rest of this paper is organized as follows. In Section 2, the study area and raw data involved in this study are introduced in detail. In Section 3, the methodology of the SSE is described, the array of input parameters is specified, and the entire ANN-based SMC retrieval process is demonstrated. In Section 4, the results are discussed regarding the retrieval accuracy improvement brought by the SSE and the respective influences of the ANN input parameters and their combinations on SMC retrieval. Finally, Section 5 presents the conclusions drawn from the study results.

## 2. Study Area and Dataset

### 2.1. Study Area and Ground Truth Data

**Figure 1:**
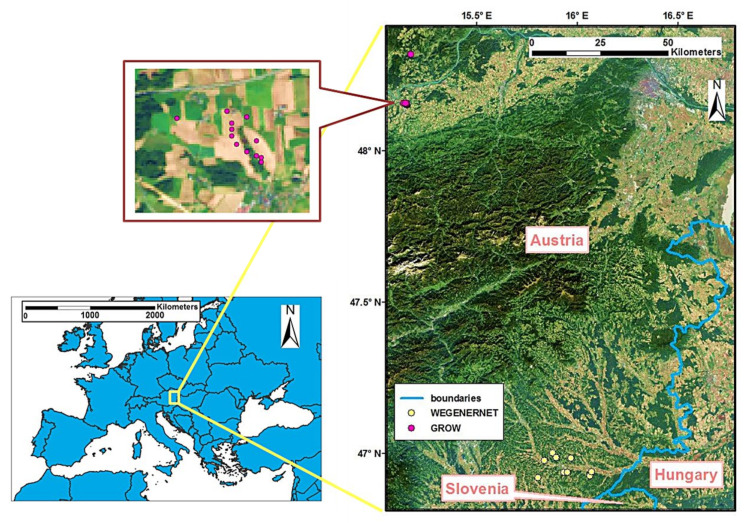
Location of the study area and monitoring sites.

The study area is located in the eastern part of Austria (Figure 1). Compared with the Eastern Alps region in the middle and west of the country, the topography in the study area is flatter, but hilly terrain still exists. The winter is often cold, but temperatures can be relatively high in summer, and the continental climate features dominate, thus, the precipitation tends to be low [38]. The ground surface is prevalently covered by vegetation, and land use types mainly comprise cropland, forest, and grassland. The croplands are rainfed, and the staple crops are wheat and corn. Closed forests feature in the study area, with the fractional vegetation cover (FVC) > 0.4. The principal tree species contain oak, hornbeam, and beech. As for the hydrological conditions, surface water in this region is closely related to the groundwater [39].

Ground truth data come from The International Soil Moisture Network (ISMN), which was implemented in 2009 aiming exclusively to validate and calibrate SMC retrieval with RS techniques. The data are qualitatively controlled after collecting them from the networks and then distributed on the website portal (https://ismn.geo.tuwien.ac.at/, accessed on 20 January 2022) [40,41]. This study selected monitoring sites from WEGENERNET and GROW, two soil moisture networks in Austria. The WEGENERNET network is situated in Styria State with nine monitoring sites, and the GROW network is located in Lower Austria State with 13 monitoring sites. WEGENERNET is a durable network with relatively continuous SMC data acquisition dating from 2007. We adopted data from January 2016 to May 2020 for our research. In contrast, for GROW, the data were available only between May 2017 and June 2019 in an intermittent manner. The SMC data at a depth of 0–5 cm was chosen considering the detecting ability of remote sensing techniques used in this study. Table 1 shows the coordinates (latitude and longitude), the network, and each site’s land cover type. As the ground truth data, the SMC observations were recorded with acquisition times in accordance with the corresponding acquisition times of the SAR images (described below).

### 2.2. Remote Sensing Data

The optical RS data employed in this study was obtained by the Landsat-8 satellite. Onboard the Landsat-8 satellite were two sensors, namely operational land imager (OLI) and thermal infrared sensor (TIRS), which help obtain multi-band data in the form of visible and infrared spectra with a fine resolution. We chose Landsat-8 images considering the synchronization of optical and land surface temperature data. The Landsat-8 images were utilized to extract optical data and calculate land surface temperature by the thermal infrared band. In this study, OLI-TIRS Level-1 images, downloaded from the United States Geological Survey (USGS) data archive (https://earthexplorer.usgs.gov/, accessed on 20 January 2022), were selected. The span period was from January 2016 to April 2020, and the spatial resolution of the images was 30 m. Based on the method described in Section 3.1, images were selected as long as they covered at least one monitoring site that was clear and without cloud obstruction on the date of image acquisition. The optical RS data were then subject to preprocessing procedures, including radiometric correction, FLAASH atmospheric correction, and band calculation. Finally, the NDVI and LST values in the monitoring sites were derived and recorded.

The microwave RS data employed in this study came from the Sentinel-1 satellite. Sentinel-1 provides VH and VV polarization modes C-band images with relatively high spatiotemporal resolution and radiometric accuracy. The imaging data played a crucial part in dynamic hydrological processes and SMC monitoring [42,43,44,45,46]. Here, the interferometric wave (IW) mode images were utilized with a spatial resolution of 10 m and a revisit period of 6 days. The images were downloaded from https://search.asf.alaska.edu/ (accessed on 20 January 2022) by courtesy of the Alaska Satellite Facility (ASF). We chose Sentinel-1 images of the study area based on their acquisition times to ensure that the radar and optical data were approximately synchronous in pairs. The temporally nearest microwave image was selected for each optical image collected already. It was confirmed that the acquisition times of the microwave images were less than five days away from the acquisition times of their optical counterparts. Furthermore, we checked the intervals between the acquisition times of each microwave image and their corresponding optical image to ensure that no precipitation event had occurred during the gaps. Subsequently, the microwave images underwent preprocessing as well. The preprocessing procedures included multi-looking, filtering, topographical correction, geocoding, and radiometric calibration. Finally, the backscattering coefficients in the VH and VV polarization modes of each monitoring site were derived, and the radar incidence angles were recorded.

Table 2 shows the acquisition times of the RS data used in this study. The dates of the radar and optical images were given in pairs. 

### 2.3. Auxiliary Data

The auxiliary data contained a digital elevation model (DEM) and land cover product. This study used DEM from Shuttle Radar Topography Mission (SRTM) downloaded from the USGS website (http://gdex.cr.usgs.gov/gdex/, accessed on 20 January 2022). The slope data were then derived from DEM using the “Slope” tool integrated into the ArcMap 10.5 software. Both the elevation and slope of each monitoring site were extracted and recorded. We obtained land cover data referring to “Global Land Cover with Fine Classification System at 30 m” (GLC_FCS30) downloaded from http://data.casearth.cn/ (accessed on 20 January 2022). The land cover types of the monitoring sites were collected. Because of the evident attenuation effect of dense vegetation canopies on C-band radar backscattering [47,48], we eliminated the monitoring sites located in the forests. Considering the subsequent operations of ANN training and testing, the land cover types were transformed into numerical data. “Cropland” and “Grassland” were substituted with “1” and “2,” respectively.

### 2.4. Sample Pool

After the processing procedures, the data were used to form a collection of samples. If one monitoring site had “complete” data on one particular date, with optical RS data, microwave RS data, auxiliary data, and in situ SMC measurement all accessible, then the corresponding sample will be assembled. Each sample can be considered a 10-dimensional vector, comprising 9 parameters derived from RS and auxiliary data and one ground-truth SMC observation (specified below in Section 3.2). The samples were placed in the sample pool (635 in aggregate) and ready to be designated as training/validation/testing datasets in the subsequent ANN training and testing processes.

## 3. Methodology

### 3.1. Sample Quantity Optimization: Sparse Sample Exploitation

In this section, the SSE method is put forward in detail. In essence, the SSE is a sort of data expansion technique over the time scale. By taking full advantage of the available images and observations, it manages to gather more samples derived over a wider time frame, thereby transferring more valuable information into the sample pool, and helping to accomplish SMC retrieval with higher precision. Briefly, the SSE involves 2 steps:

1. For dates when the sky above the study area is clear and no in situ observation is absent, all the samples are recorded in the sample pool.
2. For dates when the study area is partially blocked by clouds or in situ observations are absent, the “sparse samples” with available optical, microwave data, and ground truth observations are recorded similarly in the sample pool.

**Figure 2:**
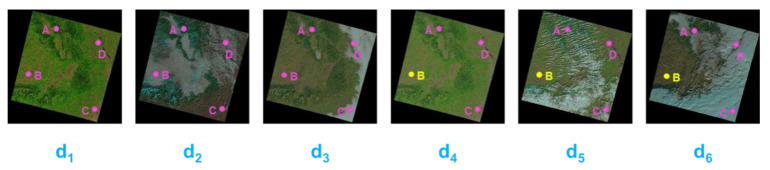
Schematic of sample collection process of SSE method.

To illustrate this method straightforwardly, we take Figure 2 as an example. In Figure 2, the images are the optical RS images covering the region of interest on six different dates, namely d_1_, d_2_, …, d_6_. Points A, B, C, and D denote the locations of the monitoring sites, of which the RS data and in situ SMC observations are expected. The points in pink indicate that in situ data are available, whereas the points in yellow indicate that in situ data are missing. 

As for d_1_, the image is cloud-free, and every monitoring site has its SMC observations; hence, the samples derived from the four sites are all valid for the sample pool. For d_2_ and d_3_, the clouds start to interfere. For the traditional sample-picking method mentioned in previous studies, data in these dates should be dismissed because optical RS data corresponding to specific sites are unavailable, and not all sites have complete data. However, it can be found that points such as C in the image of d_2_ and A and B in the image of d_3_ are clear in optical RS images and can still yield complete data. The samples corresponding to these points are designated as “sparse samples.” For the SSE method, these samples are considered to be included in the sample pool.

Similarly, for d_4_, d_5_, and d_6_, when point B has no available in situ SMC observation due to, hypothetically, instrument power failure, the data from point B are consequently eliminated. For the traditional sample-picking method, the whole data in these dates will again be abandoned due to the data’s incompleteness. For the SSE method, however, because samples can still be formed from the complete data of points A, C, D on date d_4_ and D on date d_5_, these samples are thus collected in the sample pool. On date d_6_, no sample can be collected.

Table 3 lists the comparison of sample selection via the traditional and SSE methods. It is evident that for the traditional method, the quantity of samples is severely limited, owing to the requirement of data completeness in the entire study area when collecting samples. Therefore, the samples from the four points in d_1_ will be the only valid ones. In contrast, the SSE method manages to enlarge the sample pool by making full use of the sparse samples. In this study, a total of 635 samples can be collected by the SSE method, but only 264 out of the 635 samples can be collected if the traditional method is implemented.

### 3.2. Sample Quality Optimization: Input Parameter Selection

For more accurate SMC retrieval results, the combination of inputs of the ANN is supposed to contain enough variables to represent the main features [32]. In addition to these commonly discussed parameters, including radar backscattering coefficient, radar incidence angle, and NDVI, some other SMC-related variables, such as data acquisition time, land surface temperature, elevation, slope, and the land cover type, are worth considering as well.

1. Data acquisition time: the data acquisition time was strongly correlated to the surface soil hydraulic conductivities [49]. Meanwhile, the phenological traits of vegetation follow a circulation of alteration on an annual basis [50,51], which plays an essential role in vegetation effect elimination during the process of SMC retrieval in vegetation-covered areas.
2. Land surface temperature (LST): previous studies have proven the correlation of variation between the SMC and temperature vegetation dryness index (TVDI) [52,53]. The synergy of LST and vegetation indices (such as NDVI) on SMC retrieval has also been stressed [54,55,56].
3. Elevation and slope: soil moisture was closely related to the local topographical heterogeneity. The landscape shapes physically controlled the hydrological processes and SMC time stability [57,58], with upland water moving to the groundwater and lowland water coming from the groundwater, and water content increasing from the top to the bottom of a slope in a non-linear pattern [59,60].
4. Land cover type: the land use was analyzed as a factor influencing soil hydraulic attributes and SMC distribution. For example, human activities such as grazing, plowing, and urban development impact the macropores and the continuity of the macropore network of soil, thus altering the mode of local soil water supply and SMC distribution [49,61].

By referring to these existing studies, we here selected 9 parameters derived from the RS and auxiliary data, namely the data acquisition time (month), radar incidence angle (*θ*), VH backscattering coefficient (*σ*_VH_), VV backscattering coefficient (*σ*_VV_), NDVI, LST, elevation, slope, and land cover type, as inputs of the ANN. The acquisition of the parameters was explained in the previous sections, and we introduced the ordinal number of the data acquisition month to present the data acquisition time for the ANN calculations.

Furthermore, to investigate the effects of the input parameters and their combinations on SMC retrieval, a total of 7 scenarios were considered, as shown in Table 4. In Scenario 0, all the 9 parameters were taken into account; in Scenario 1, the 4 commonly discussed parameters, i.e., *θ*, *σ*_VH_, *σ*_VV,_ and NDVI, were included as the basic inputs; in Scenario 2–6, the other 5 parameters were added individually into the basic input parameters. By comparing the SMC retrieving results of these scenarios, the sensitivity of SMC to specific input parameters was assessed and analyzed.

### 3.3. ANN and SMC Retrieval

After selecting data using the SSE method and determining input parameters, a group of samples was obtained. The ANN approach was then adopted to retrieve the SMC. The ANN is the abstraction of the neural network of human brains from the perspective of data processing. With the nodes of neurons connected sequentially, the ANN is organized into a layered structure. As the data are inputted to the ANN, neurons perform weighted computations and pass on the results to other neurons until reaching the output layer, which yields the final result [31,62]. In terms of SMC estimation, the ANN approach provides a better solution than conventional theoretical and semi-empirical models owing to its capacity for describing non-linear relationships [63].

**Figure 3:**
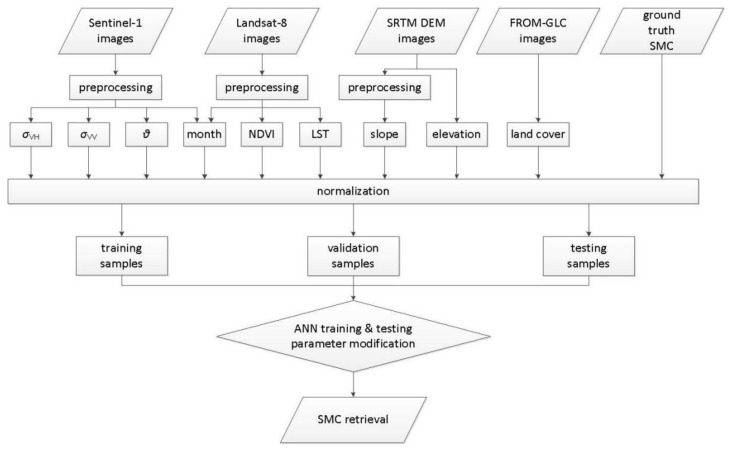
Flowchart of the SMC retrieval by ANN.

Moreover, the independence of the ANN from a priori knowledge and radiative transfer information relieves the estimation process of explicit physical mechanism and complicated parameters, and the parameters or combinations involved can be more flexible [64,65]. Figure 3 shows the flowchart of the SMC retrieval process developed in this study, with all 9 input parameters mentioned above utilized. Here, a feed-forward perceptron model was employed, and the ANN has a 3-layer structure comprising input, hidden, and output layers.

The number of neurons in the hidden layer is another essential characteristic. Too few or too many neurons may lead to underfitting or overfitting, thus affecting the accuracy of SMC retrieval [66]. In this study, 10 neurons were contained in the hidden layer, determined through a trial-and-error method. The SMC ground-truth observations were set as outputs. Next, both the inputs and outputs were normalized to 0 to 1 based on their respective range of values. The normalization procedure can improve the training speed and help prevent the outcomes from getting stuck in local minimums to a certain extent [66].

The samples in the sample pool were randomly partitioned into training, validation, and testing datasets in proportions of 80%, 10%, and 10%, respectively, for the following ANN training and testing. The purpose of ANN training was to iteratively modify the weights of correlation between the inputs and outputs thus that the differences can be minimized. The training process was accomplished with training samples as well as validation samples. The validation samples here were aimed at ensuring the generalization capacity of the ANN and avoiding overfitting during the training process [64]. The Levenberg–Marquardt method was chosen as the training algorithm. This method provides an optimal solution for a certain minimizing problem [67]. Numerous iterations were conducted in search of an optimal solution during the training process, and the maximum number of iterations was set as 1000. The training process was stopped either when the generalization capacity of the ANN began to level off, which indicates that more training processes cannot improve the accuracy, or when the maximum number of iterations was reached. The testing process was performed with the testing samples by comparing the ground truth SMC with the estimated SMC derived from the corresponding inputs using the trained ANN. The training and testing processes were conducted in MATLAB, and the well-trained ANN was deployed for SMC mapping in the entire study area.

### 3.4. Statistical Metrics

The retrieval accuracy was evaluated using 2 statistical metrics: the root-mean-square error (RMSE) and the correlation coefficient (*r*), which can be expressed as follows:

(1) $$\mathrm{RMSE}=\sqrt{\frac{1}{n}\overset{n}{\underset{i=1}{\sum }}{\left(SMC_{i}-{\hat{SMC}}_{i}\right)}^{2}}$$

(2) $$r=\frac{\sum_{i=1}^{n}\left(SMC_{i}-\bar{SMC}\right)\left({\hat{SMC}}_{i}-\bar{\hat{SMC}}\right)}{\sqrt{\sum_{i=1}^{n}{\left(SMC_{i}-\bar{SMC}\right)}^{2}}\sqrt{\sum_{i=1}^{n}{\left({\hat{SMC}}_{i}-\bar{\hat{SMC}}\right)}^{2}}}$$

where $SMC_{i}$ and $\bar{SMC}$ represent the *i*th sample’s ground-truth and mean ground-truth SMC values of the relevant samples; ${\hat{SMC}}_{i}$ and $\bar{\hat{SMC}}$ represent the *i*th sample’s estimated SMC value and the mean estimated SMC values of all the relevant samples, respectively. The RMSE and *r* were calculated based on the training, validation, and testing results.

## 4. Results and Discussion

### 4.1. Evaluation of Overall Accuracy 

**Figure 4:**
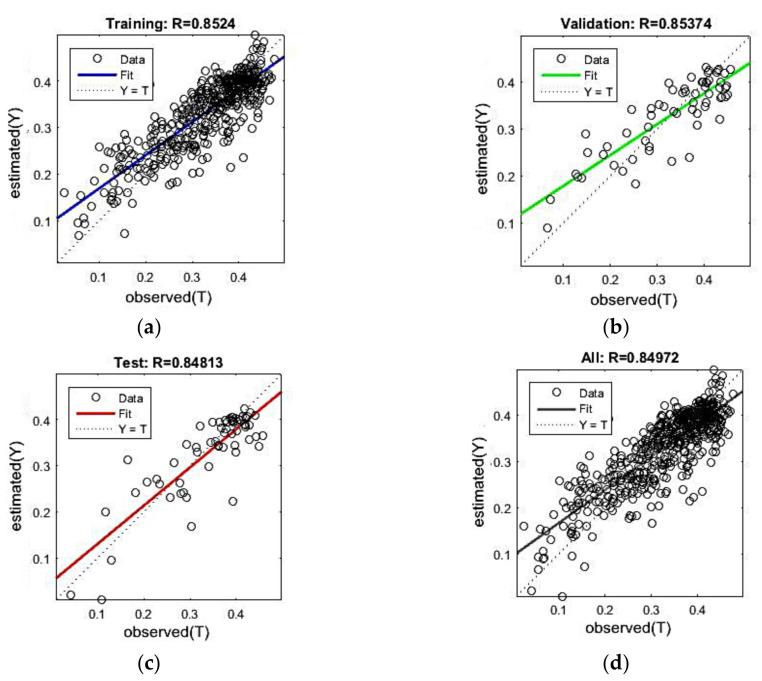
Scatter plots of SMC estimations for training (**a**), validation (**b**), testing dataset (**c**), and the entire samples (**d**). Corresponding correlation coefficients are placed above each plot.

First, the overall accuracy of the ANN was evaluated. All the 635 samples were used and divided into training, validation, and testing datasets. The training/testing process was conducted once, with all nine parameters being involved as the inputs of the ANN (Scenario 0 in Section 3.2). Figure 4 shows the scatter plots of the SMC estimation results for the training, validation, testing datasets, and the entire samples. The correlation coefficient (*r*) values were also given above each plot. The results were promising, with the testing dataset *r* and overall *r* reaching 0.85. Table 5 shows the corresponding RMSE values, which seem favorable, with RMSE values of 0.048 m^3^m^−3^, 0.054 m^3^m^−3^, and 0.052 m^3^m^−3^ on the training, validation, and testing datasets, respectively. The ground-truth SMC values range from 0.024 to 0.477 m^3^m^−3^ with an average value of 0.336 m^3^m^−3^, whereas the estimated SMC values ranged from 0.039 to 0.470 m^3^m^−3^ with the average value of 0.335 m^3^m^−3^. In comparison with the work conducted by Alexakis et al. [33], our study quantitatively expanded the sample pool and qualitatively improved the accuracy of SMC retrieval, with the testing dataset *r* rising from 0.508 to 0.848 and the overall *r* rising from 0.803 to 0.850.

### 4.2. Evaluation of SSE Method

The effectiveness of the proposed SSE method was evaluated by comparing the SMC retrieval results with and without sparse sample exploitation. Concerning the study by Holtgrave et al. [68], considering that different schemes of the random division of training/validation/testing datasets might give rise to different SMC retrieval outcomes, we repeated the ANN training/testing process 50 times to assess the average SMC retrieval performance. Since the accuracy of the testing datasets was more worthwhile in terms of the ANN performance, only the statistical metrics on the testing datasets were discussed in the remainder of the paper. Similarly, all nine parameters were set as the inputs of the ANN (Scenario 0 in Section 3.2). Table 6 lists the average RMSE and *r* values on the testing datasets for SMC retrieval with and without the SSE method.

The results indicate a striking increase in the SMC retrieval accuracy when introducing the SSE method, with the RMSE decreasing from 0.090 m^3^m^−3^ to 0.068 m^3^m^−3^ and *r* increasing from 0.635 to 0.736. The main reason could be the efficient utilization of the dismissed samples in the images where optical or in situ data were partially missing, and the sample pool could consequently be expanded. For the empirical SMC retrieval methods, such as ANN, large samples were required during the training process. Thus, the precise relationship between the inputs and outputs could be established [69]. Although the SSE did not conventionally ensure that all the monitoring sites had identical time series of data acquisition, it nonetheless enlarged the sample capacity by gathering more samples derived over a broader period. Meanwhile, the information provided by these samples could be made full use of, and the features of the training dataset were enriched, consequently enhancing the representativeness of the samples as well as the stability of the ANN. Therefore, the training precision of the ANN was improved.

### 4.3. Sensitivity Analysis of Input Parameters

As mentioned in Section 3.2, several scenarios were considered for the sensitivity analysis of different input parameters. Table 7 shows scenarios 1–6 of input parameter combinations and their statistical metrics for SMC retrieval. During the sensitivity analysis, all the 635 samples were employed. Similarly, the ANN training/testing process was repeated 50 times after random divisions of each scenario’s training/validation/testing datasets. The mean statistical metrics on the testing dataset representing the average performances were evaluated. As listed in Table 7, the first scenario was the combination of basic input parameters, including the VH/VV backscattering coefficients, NDVI, and radar incidence angle. For the rest of the scenarios, the data acquisition time, LST, elevation, slope, and land cover were added individually to the basic combination.

#### 4.3.1. Data Acquisition Time

Comparing scenarios 1 and 2: after the data acquisition time (i.e., the data acquisition month in this study) was added as the input parameter, *r* increased from 0.588 to 0.637, and RMSE declined from 0.089 m^3^m^−3^ to 0.078 m^3^m^−3^.

The influences of adding data acquisition time on each sample were investigated for further analysis. For each sample participating in SMC retrieval, “accuracy improvement.” $I_{i}$ was proposed with the expressions below:

(3) $$I_{i}=\varepsilon_{i_{\mathrm{basic}}}-\varepsilon_{i_{\mathrm{new}}}$$

(4) $$\varepsilon_{i\_\mathrm{basic}}=\frac{\left|SMC_{i}-SMC_{i\_\mathrm{basic}}\right|}{SMC_{i}}\times 100\%$$

(5) $$\varepsilon_{i\_\mathrm{new}}=\frac{\left|SMC_{i}-SMC_{i\_\mathrm{new}}\right|}{SMC_{i}}\times 100\%$$

where $SMC_{i}$ denotes the ground-truth SMC of the *i*th sample, $SMC_{i\_\mathrm{basic}}$ denotes the estimated SMC of the *i*th sample with only basic input parameters as the ANN inputs, and $SMC_{i\_\mathrm{new}}$ denotes the estimated SMC of the *i*th sample with a new parameter incorporated into the basic ones as the ANN inputs. $\varepsilon_{i\_\mathrm{basic}}$ and $\varepsilon_{i\_\mathrm{new}}$ denote the corresponding relative errors of the *i*th sample. $I_{i}$, the value of accuracy improvement is the difference between the two errors. When $I_{i}$ is positive, it means that the error of SMC retrieval with the new input parameter is lower than that of SMC retrieval by basic input parameters, indicating a real accuracy improvement of SMC retrieval; conversely, when $I_{i}$ is negative, it means adding the new input parameter in the ANN brings about a worse result. In addition, $\bar{I}$ was used to denote the average accuracy improvement of corresponding *i* samples:

(6) $$\bar{I}=\underset{i}{\sum }I_{i}$$

Table 8 lists the results of accuracy improvement of SMC retrieval by adding data acquisition time as the input parameter. Because of the distinctive phenological pattern of cropland, we paid extra attention to the cropland samples. For all 635 samples, the number of samples with $I_{i}>0$ reached 372. Among these samples, 287 were cropland samples, accounting for 77.2%. As for $\bar{I}$, the average accuracy improvement of all samples was 6.64%, whereas for cropland samples, the $\bar{I}$ was 5.66%, accounting for 85.2% of the total gain. These results indicate that the addition of data acquisition time as the input parameter improves the SMC retrieval performance of cropland samples, thus driving up the retrieving accuracy of the entire samples.

**Figure 5:**
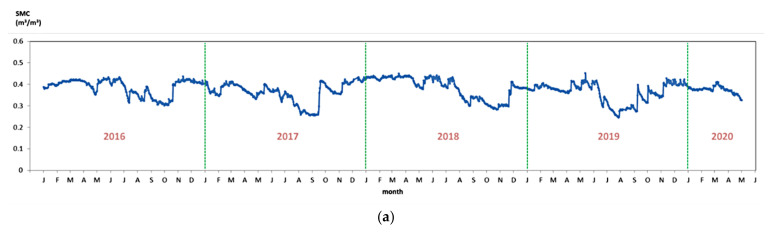

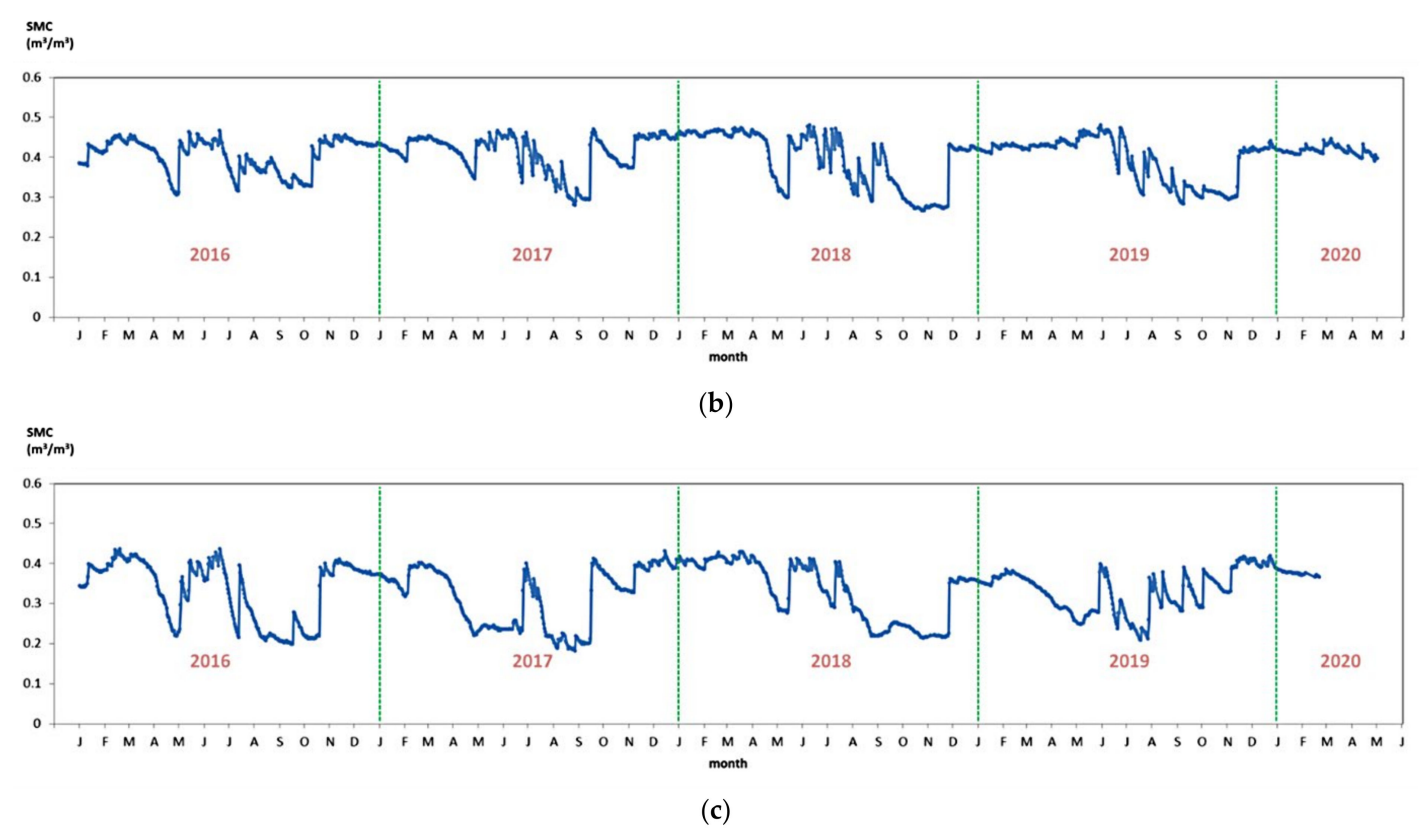
Ground-truth SMC time series of some monitoring sites: (**a**) Site #5 (cropland), (**b**) Site #7 (grassland), (**c**) Site #9 (cropland).

In fact, the season or data acquisition time was strongly correlated to the plant growth condition and the corresponding SMC ground-truth data in the vegetation-covered regions. Here, we chose three monitoring sites of which the SMC observations were continuous and long-lasting, and the SMC time series are displayed in Figure 5. These SMC time series generally present a periodic pattern of annual variation, respectively. For site #5, SMC observations are high in winter and spring, begin to fluctuate in summer and keep relatively low in August and September. For site #7, the fluctuations in summer were more drastic, and sharp declines occurred around May in three consecutive years (2016, 2017, and 2018). For site #9, the SMC variation is not so regular; however, some annual patterns, such as the plateaus in February and March, the significant dips after summer, and the rises in November, are still observable.

Hence, as an input parameter, the data acquisition time contributes to a more delicate description of the vegetation phenological features, and better SMC retrieval outcomes can thus be obtained.

#### 4.3.2. LST

Comparing scenarios 1 and 3: The addition of LST helped increase *r* to 0.616 and decrease the RMSE to 0.084 m^3^m^−3^.

Sandholt et al. [53] defined the temperature vegetation dryness index (TVDI) as:

(7) $$TVDI=\frac{LST-LST_{\min}}{LST_{\max}-LST_{\min}}$$

where $LST_{\min}$ and $LST_{\max}$ are the minimum and the maximum land surface temperatures, respectively, corresponding to a specific NDVI value in the LST-NDVI space. The correlation of TVDI and SMC suggests the rationality of SMC retrieval with the synergy of NDVI and LST. 

**Figure 6:**
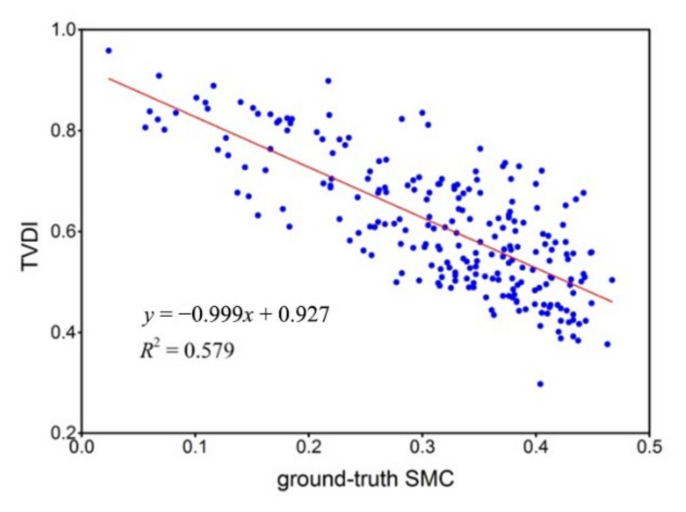
Scatter plot of the relationship between TVDI and ground-truth SMC for those samples with positive $I_{i}$ after the addition of LST as the input parameter.

After the addition of LST as the input parameter, for those samples with positive $I_{i}$, the scatter plot of the relationship between TVDI and ground-truth SMC is shown in Figure 6, and the negative correlation is evident.

Furthermore, Schmugge [70] claimed that the soil’s surface temperature was the function of both internal and external factors. The thermal conductivity and heat capacity, which belonged to the internal factors, both increased with the rise of SMC. As a factor reflecting the intensity of evapotranspiration, the spatial distribution of the LST varied significantly with the land surface water. 

In this study, the ANN managed to retrieve the SMC with higher accuracy with the aid of the LST. This result was further proof for the conclusions made in the studies mentioned above in Section 1. 

#### 4.3.3. Elevation and Slope

Comparing scenarios 1, 4, and 5: the retrieval accuracy improved remarkably after incorporating the elevation into the input parameter pool. The *r*-value increased to 0.689, and the RMSE decreased to 0.070 m^3^m^−3^. The slope promoted accuracy, with *r* up to 0.639 and RMSE falling to 0.083 m^3^m^−3^. 

**Figure 7:**
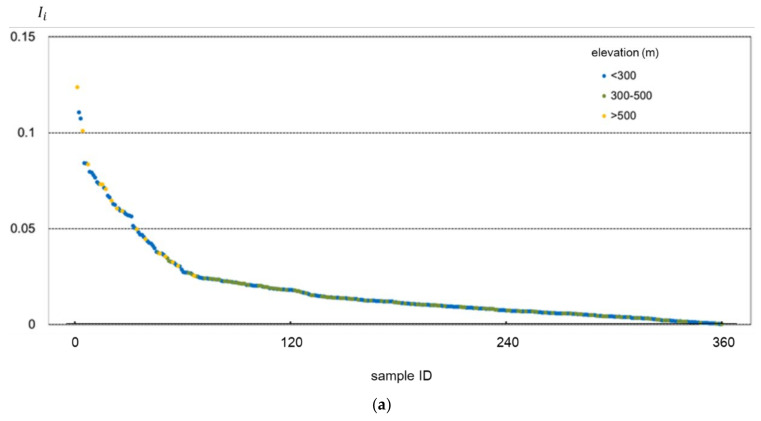

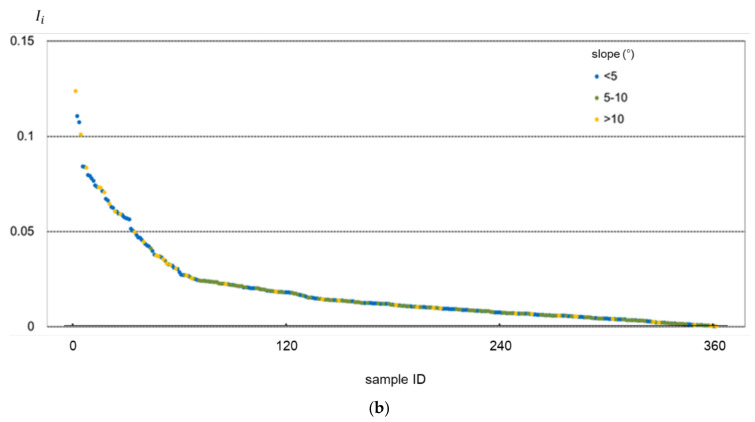
The accuracy improvement of samples by adding elevation as the input parameter. In (**a**), the samples are categorized into three groups by elevation and (**b**) by the slope.

**Figure 8:**
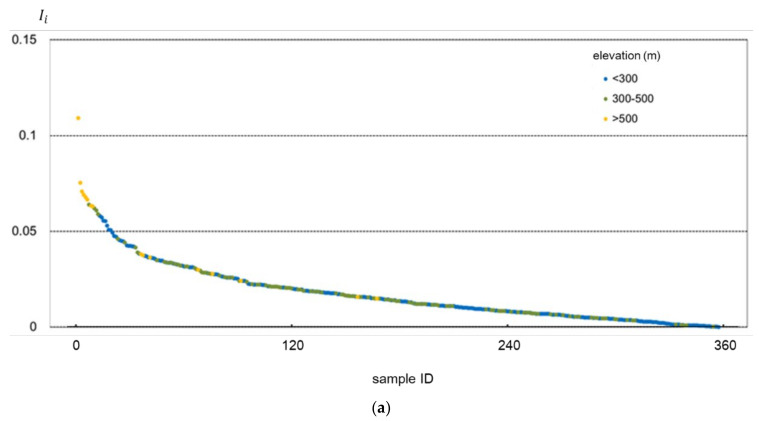

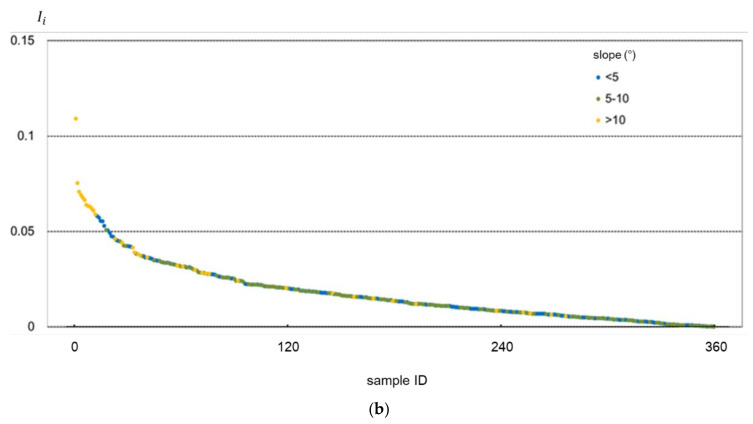
The accuracy improvement of samples by adding slope as the input parameter. In (**a**), the samples are categorized into three groups by elevation and (**b**) by the slope.

For further explanation, the accuracy improvement of those samples improving SMC retrieval accuracy ($I_{i}>0$) by virtue of incorporating topographic factors is illustrated in Figure 7 and Figure 8. Figure 7 displays the accuracy improvement by adding elevation as the input parameter for different samples of elevation and slope values. In contrast, Figure 8 indicates the accuracy improvement by adding slope as the input parameter in relation to samples of different elevation and slope values. In each figure, samples are arranged in descending order of their corresponding *I_i_*

It can be observed from Figure 7 and Figure 8 that, no matter for adding elevation or adding slope as the input parameter, samples with relatively higher elevation (>500 m) and steeper slope (>10°) tended to yield better accuracy improvement results, with most of the corresponding samples gathering in the left of the figures. 

Previous studies claimed that local topographical heterogeneity reinforced the variation in the soil moisture distribution. Due to gravity and overland flow, locations with a high elevation and steep slope were more prone to SMC change. In contrast, low and flat locations were more inclined to SMC invariability [57,58,60,71]. Analogous to these studies, the difference in the topography of our study area was noticeable enough to impact the soil moisture distribution as well. Therefore, taking the elevation and slope into consideration during SMC retrieval was rational.

#### 4.3.4. Land Cover Type

Comparing scenarios 1 and 6: when land cover type was considered an input parameter, the accuracy of SMC retrieval failed to improve as expected. Despite the existing studies emphasizing the influences of land use on SMC distribution [72,73], the outcome of ANN-based SMC retrieval with the assistance of land cover type did not show any improvement. The *r* slightly increased to 0.599, whereas the RMSE rose marginally to 0.091 m^3^m^−3^. This was probably attributed to the poor land cover categorization of the samples. In this study, after eliminating forest, the samples merely fell into two land cover types; in practice, the ground-truth geographical conditions of the study area could be quite intricate. The land cover categorization could not adequately improve the accuracy of SMC retrieval, and a refined land cover map was required.

### 4.4. SMC Mapping

**Figure 9:**
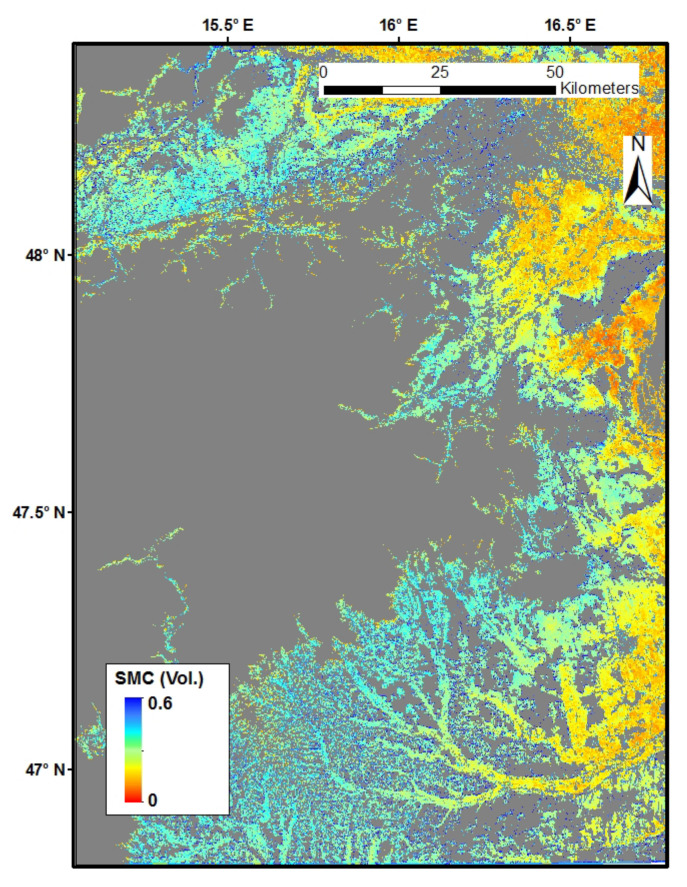
Volumetric SMC mapping of the study area.

Figure 9 shows the map of the SMC retrieval outcome at a depth of 5 cm for the study area on 6 October 2018. Considering the representativeness of the training samples, regions of forests and high elevation (>500 m) were masked. In addition, the water bodies and residential areas where no soil existed were masked as well. The gray patches indicate masked regions. The soil moisture distribution was visually plausible based on the map, with shades of blue and green (high SMC) mainly representing cropland and grassland, while red or yellow ones (low SMC) representing relatively bare land.

## 5. Conclusions

An ANN approach for SMC retrieval using microwave RS data (Sentinel-1 SAR images) and optical RS data (Landsat-8 images) was demonstrated, and a novel SSE methodology was proposed. With the SSE, the problem of data deficiency due to cloud contamination in optical images and in situ instrument malfunction was resolved. Complete data were fully utilized in the ANN training/testing procedure, and the enlarged sample pool was beneficial to SMC retrieval with high precision. 

The sample volume could be increased from 264 to 635 by the SSE, and the SMC retrieval accuracy was significantly enhanced. Regarding the average statistical metrics corresponding to 50 ANN training/testing iterations, *r* increased from 0.635 to 0.736, and the RMSE decreased from 0.090 m^3^m^−3^ to 0.068 m^3^m^−3^. 

A couple of variables were newly considered about the inputs of ANN for SMC retrieval. As for the sensitivity analysis of the ANN inputs, the parameters, such as the elevation, slope, data acquisition time, LST, and the land cover type, influenced the SMC retrieval accuracy to varying degrees. Among these parameters, the elevation had the most significant impact on the results, as evidenced by the increase in the *r*-value from 0.588 to 0.689 and the decrease in the RMSE from 0.089 m^3^m^−3^ to 0.070 m^3^m^−3^. Other parameters were also advantageous to SMC retrieval, except for the land cover type, which barely promoted the accuracy due to the lack of refined land cover categorization. Notably, overall, the SMC retrieval statistical metrics of Scenario 0, where all nine relevant input parameters were considered (the situation “with SSE” discussed in Section 4.2), proved to be much more favorable than those of the scenarios analyzed in Section 4.3. This signifies that, to some degree, more relevant input parameters tend to improve retrieval accuracy.

The study results show that SSE is a promising method for ANN-based SMC retrieval. However, certain limitations need to be addressed. Because study areas overseas are beyond our reach and field surveys on topography and land cover are challenging to implement, the inconsistency between ground truth data and RS data cannot be excluded. The consequent biases in the SMC retrieval are inevitable. Moreover, the SMC mapping lacks additional in situ data for further validation.

We plan to focus on sample exploitation over the spatial dimension in the future. In other words, for study areas without sufficient samples for ANN training, synchronous data from another site of geographical resemblance with sufficient samples will be considered for SMC retrieval. The accuracy and conditions for the application of this method will be investigated. Additionally, we intend to utilize state-of-the-art RS data from Chinese satellites, such as GF-3 and GF-1, and explore their applicability in SMC retrieval problems.

## Figures and Tables

**Table 1:** Information of monitoring sites in the study area.

#	Lat. and Long.	Network	Landcover	#	Lat. and Long.	Network	Landcover
1	46.91691° N 15.78112° E	WEGENERNET	farmland	12	48.15202° N 15.15303° E	GROW	farmland
2	46.97232° N 15.81499° E	WEGENERNET	farmland	13	48.15257° N 15.15104° E	GROW	farmland
3	46.99726° N 15.85507° E	WEGENERNET	farmland	14	48.15356° N 15.14857° E	GROW	farmland
4	46.98299° N 15.87115° E	WEGENERNET	farmland	15	48.15403° N 15.15299° E	GROW	farmland
5	46.93296° N 15.90710° E	WEGENERNET	farmland	16	48.15474° N 15.14844° E	GROW	farmland
6	46.93291° N 15.92462° E	WEGENERNET	grassland	17	48.15562° N 15.14804° E	GROW	farmland
7	46.97970° N 15.94122° E	WEGENERNET	grassland	18	48.15645° N 15.14799° E	GROW	farmland
8	46.92135° N 16.03337° E	WEGENERNET	farmland	19	48.15709° N 15.13658° E	GROW	farmland
9	46.93427° N 16.04056° E	WEGENERNET	farmland	20	48.15725° N 15.15149° E	GROW	farmland
10	48.15117° N 15.15417° E	GROW	farmland	21	48.15804° N 15.14731° E	GROW	farmland
11	48.15179° N 15.15424° E	GROW	farmland	22	48.18776° N 15.98071° E	GROW	grassland

**Table 2:** Acquisition times of RS images used in the study.

#	Dates of Radar Images	Dates of Optical Images	#	Dates of Radar Images	Dates of Optical Images	#	Dates of Radar Images	Dates of Optical Images
1	18 January 2016	18 January 2016	24	24 June 2017	22 June 2017	47	3 February 2019	4 February 2019
2	26 January 2016	27 January 2016	25	31 July 2017	31 July 2017	48	27 February 2019	27 February 2019
3	30 March 2016	31 March 2016	26	11 August 2017	9 August 2017	49	23 March 2019	24 March 2019
4	18 April 2016	16 April 2016	27	4 November 2017	4 November 2017	50	30 March 2019	31 March 2019
5	23 April 2016	23 April 2016	28	20 November 2017	20 November 2017	51	16 April 2019	16 April 2019
6	4 July 2016	5 July 2016	29	5 December 2017	6 December 2017	52	27 April 2019	25 April 2019
7	12 July 2016	12 July 2016	30	24 February 2018	24 February 2018	53	2 May 2019	2 May 2019
8	23 July 2016	21 July 2016	31	21 April 2018	22 April 2018	54	18 May 2019	18 May 2019
9	29 August 2016	29 August 2016	32	28 April 2018	29 April 2018	55	3 June 2019	3 June 2019
10	22 September 2016	23 September 2016	33	31 May 2018	31 May 2018	56	14 June 2019	12 June 2019
11	29 September 2016	30 September 2016	34	2 July 2018	2 July 2018	57	19 June 2019	19 June 2019
12	16 October 2016	16 October 2016	35	18 July 2018	18 July 2018	58	27 June 2019	28 June 2019
13	1 November 2016	1 November 2016	36	26 July 2018	27 July 2018	59	4 July 2019	5 July 2019
14	9 November 2016	10 November 2016	37	2 August 2018	3 August 2018	60	14 August 2019	15 August 2019
15	3 December 2016	3 December 2016	38	19 August 2018	19 August 2018	61	2 September 2019	31 August 2019
16	14 December 2016	12 December 2016	39	30 August 2018	28 August 2018	62	8 October 2019	9 October 2019
17	20 January 2017	20 January 2017	40	19 September 2018	20 September 2018	63	20 October 2019	18 October 2019
18	5 February 2017	5 February 2017	41	28 September 2018	29 September 2018	64	1 November 2019	25 October 2019
19	9 March 2017	9 March 2017	42	6 October 2018	6 October 2018	65	5 January 2020	6 January 2020
20	2 April 2017	3 April 2017	43	22 October 2018	22 October 2018	66	9 March 2020	10 March 2020
21	9 April 2017	10 April 2017	44	30 October 2018	31 October 2018	67	2 April 2020	2 April 2020
22	27 May 2017	28 May 2017	45	11 November 2018	7 November 2018	68	10 April 2020	11 April 2020
23	13 June 2017	13 June 2017	46	15 November 2018	16 November 2018	69	26 April 2020	27 April 2020

**Table 3:** Comparison of samples selection via traditional method and SSE method based on Figure 2.

Date	d_1_	d_2_	d_3_	d_4_	d_5_	d_6_
traditional method	ABCD	-	-	-	-	-
SSE method	ABCD	C	AB	ACD	D	-

**Table 4:** Scenarios of input parameter combinations for ANN SMC retrieval.

Scenario	Input Parameters
0	*θ*, *σ*_VH_, *σ*_VV_, NDVI, month, LST, elevation, slope, land cover
1	*θ*, *σ*_VH_, *σ*_VV_, NDVI
2	*θ*, *σ*_VH_, *σ*_VV_, NDVI, month
3	*θ*, *σ*_VH_, *σ*_VV_, NDVI, LST
4	*θ*, *σ*_VH_, *σ*_VV_, NDVI, elevation
5	*θ*, *σ*_VH_, *σ*_VV_, NDVI, slope
6	*θ*, *σ*_VH_, *σ*_VV_, NDVI, land cover

**Table 5:** RMSE values on training, validation, and testing datasets.

Dataset	Training	Validation	Testing
RMSE (m^3^m^−3^)	0.048	0.054	0.052

**Table 6:** Statistical metrics on testing dataset for SMC retrieval with and without the SSE method.

	Without SSE	With SSE
RMSE (m^3^m^−3^)	0.090	0.068
*r*	0.635	0.736

**Table 7:** Scenarios of different input parameter combinations and corresponding performances of SMC retrieval. Ticks indicate that the parameters are chosen as input scenarios systems.

Scenarios	Input Parameters									Statistical Metrics	
	Month	*σ*_VH_	*σ*_VV_	NDVI	LST	Elevation	Slope	Land Cover	*θ*	RMSE (m^3^m^−3^)	*r*
1		√	√	√					√	0.089	0.588
2	√	√	√	√					√	0.078	0.637
3		√	√	√	√				√	0.084	0.616
4		√	√	√		√			√	0.070	0.689
5		√	√	√			√		√	0.083	0.639
6		√	√	√				√	√	0.091	0.599

**Table 8:** Accuracy improvement by adding data acquisition time over total samples and cropland samples.

	Of All Samples	Of Cropland Samples	Percentage of Cropland Samples
$\mathrm{number}\;\mathrm{of}\;\mathrm{samples}\;\mathrm{with}\;I_{i}>0$	372	287	77.2%
$\bar{I}$	6.64%	5.66%	85.2%

## Data Availability

The data that support the findings of this study are available from the corresponding author upon reasonable request.
